# Supplementary material for: Splice-Junction-Based Mapping of Alternative Isoforms in the Human Proteome
Source: Cell Rep. Author manuscript; Available in PMC 2020 Jan 15. (PMC6961840; doi:10.1016/j.celrep.2019.11.026)
Supplement: 2 [file NIHMS1546469-supplement-2.zip › DF1/PXD010154/Spleen/Spleen_13_ILF3_WLTPVIPALWEAEAGESLELR.pdf]

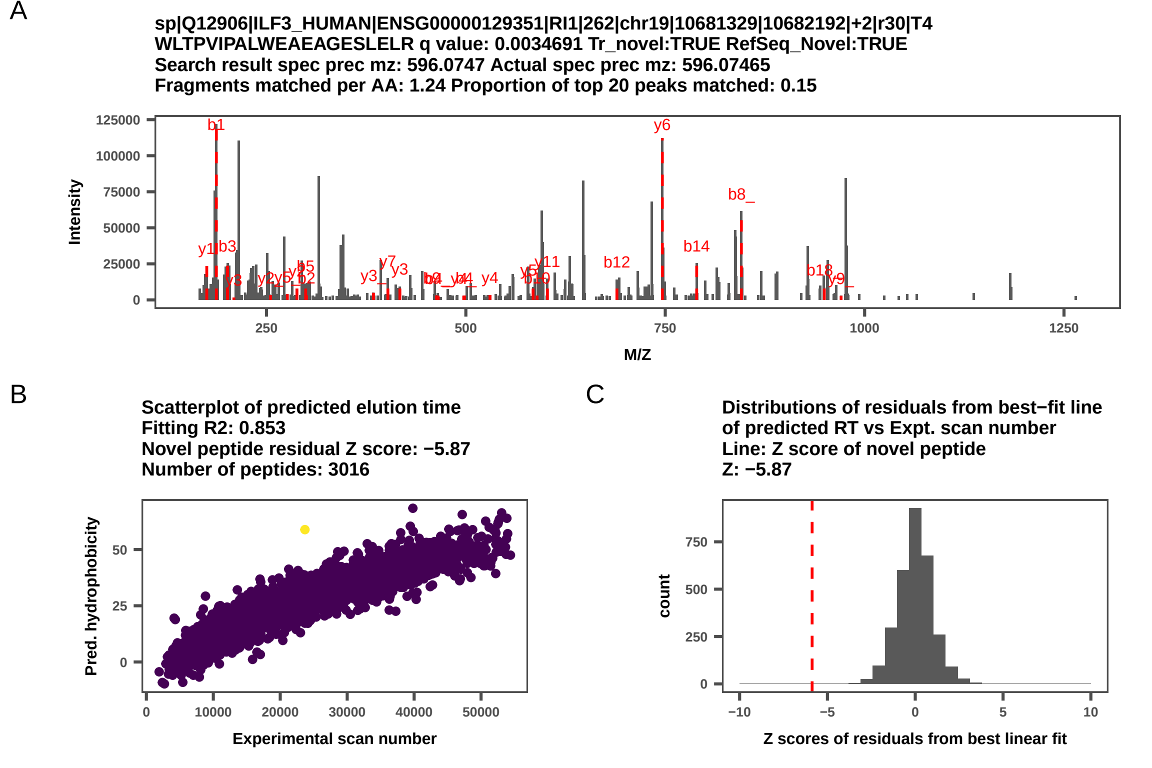
A
sp|Q12906|ILF3_HUMAN|ENSG00000129351|RI1|262|chr19|10681329|10682192|+2|r30|T4
WLTPVIPALWEAEAGESLELR q value: 0.0034691 Tr_novel:TRUE RefSeq_Novel:TRUE
Search result spec prec mz: 596.0747 Actual spec prec mz: 596.07465
Fragments matched per AA: 1.24 Proportion of top 20 peaks matched: 0.15
Intensity
125000
100000
75000
50000
25000
0
b1
y6
b8_
b14
b12
M/Z
250
500
750
1000
1250
B
Scatterplot of predicted elution time
Fitting R2: 0.853
Novel peptide residual Z score: −5.87
Number of peptides: 3016
Pred. hydrophobicity
50
25
0
0
10000
20000
30000
40000
50000
Experimental scan number
C
Distributions of residuals from best−fit line
of predicted RT vs Expt. scan number
Line: Z score of novel peptide
Z: −5.87
count
750
500
250
0
−10
−5
0
5
10
Z scores of residuals from best linear fit
